# Public Awareness of Uterine Power Morcellation Through US Food and Drug Administration Communications: Analysis of Google Trends Search Term Patterns

**DOI:** 10.2196/publichealth.9913

**Published:** 2018-04-26

**Authors:** Lauren N Wood, Juzar Jamnagerwalla, Melissa A Markowitz, D Joseph Thum, Philip McCarty, Andrew R Medendorp, Shlomo Raz, Ja-Hong Kim

**Affiliations:** ^1^ Department of Urology University of California, Los Angeles Los Angeles, CA United States; ^2^ Division of Urology Cedars-Sinai Medical Center Los Angeles, CA United States; ^3^ University of California, Los Angeles Los Angeles, CA United States

**Keywords:** Google, internet search activity, FDA safety communication, uterine morcellation

## Abstract

**Background:**

Uterine power morcellation, where the uterus is shred into smaller pieces, is a widely used technique for removal of uterine specimens in patients undergoing minimally invasive abdominal hysterectomy or myomectomy. Complications related to power morcellation of uterine specimens led to US Food and Drug Administration (FDA) communications in 2014 ultimately recommending against the use of power morcellation for women undergoing minimally invasive hysterectomy. Subsequently, practitioners drastically decreased the use of morcellation.

**Objective:**

We aimed to determine the effect of increased patient awareness on the decrease in use of the morcellator. Google Trends is a public tool that provides data on temporal patterns of search terms, and we correlated this data with the timing of the FDA communication.

**Methods:**

Weekly relative search volume (RSV) was obtained from Google Trends using the term “morcellation.” Higher RSV corresponds to increases in weekly search volume. Search volumes were divided into 3 groups: the 2 years prior to the FDA communication, a 1-year period following, and thereafter, with the distribution of the weekly RSV over the 3 periods tested using 1-way analysis of variance. Additionally, we analyzed the total number of websites containing the term “morcellation” over this time.

**Results:**

The mean RSV prior to the FDA communication was 12.0 (SD 15.8), with the RSV being 60.3 (SD 24.7) in the 1-year after and 19.3 (SD 5.2) thereafter (*P*<.001). The mean number of webpages containing the term “morcellation” in 2011 was 10,800, rising to 18,800 during 2014 and 36,200 in 2017.

**Conclusions:**

Google search activity about morcellation of uterine specimens increased significantly after the FDA communications. This trend indicates an increased public awareness regarding morcellation and its complications. More extensive preoperative counseling and alteration of surgical technique and clinician practice may be necessary.

## Introduction

Uterine power morcellation, where the uterus is shred into smaller pieces, is a widely used technique for removal of uterine specimens in patients undergoing minimally invasive abdominal hysterectomy or myomectomy [1]. The power morcellator has also been used by many practitioners during hysterectomy for women undergoing concomitant prolapse repair [2]. Morcellation was a seemingly attractive option to minimize the size of incision needed to remove a uterine specimen and decrease postoperative pain, length of hospital stay, and potential risk of hernia.

In 2014, the US Food and Drug Administration (FDA) published two safety communications regarding uterine power morcellation. The first communication, released on April 17, 2014, specifically discouraged the use of laparoscopic power morcellation during hysterectomy or myomectomy of uterine fibroids due to a small risk of spreading undiagnosed uterine sarcoma, despite the low risk of finding an unsuspected uterine malignancy [3]. On November 24, 2014, a second FDA communication was released, this time warning against the use of laparoscopic power morcellation in the majority of women undergoing treatment of fibroids with either a myomectomy or hysterectomy, again citing the low risk of spread of undiagnosed uterine sarcoma as the rationale [4]. Furthermore, practitioners were urged to communicate this information directly with patients. Subsequently, the use of the morcellator drastically decreased [5].

Previous studies have shown that public awareness of health-related spectacles can be demonstrated using Google Trends, a free publicly available tool that provides data on temporal patterns of search terms [6,7]. Google Trends can be used for causal inference, description, or surveillance of various health-related research topics [6]. We hypothesized that Google Trends may be used to determine public interest in uterine power morcellation by correlating trends with the timing of the FDA warning.

## Methods

Google Trends is a free publicly available tool that provides data on the number of times a certain term is searched over time on the internet search engine Google. We used it to examine public awareness regarding uterine power morcellation. Data are normalized to a reference population and then scaled to create a weekly relative search volume (RSV) ranging from 0 to 100, with the highest search activity scored at 100 and search activity at all other times scored relative to that peak [7]. A higher RSV represents an increase in search volume compared to other time frames, with an RSV of 100 representing a maximum search volume over a given temporal period.

We performed a Google Trends search using the term “uterine morcellation” to obtain RSVs averaged over 7-day periods. We then compared the weekly RSVs before the initial FDA communication in 2014, 1 year after the FDA warning, and thereafter. Analysis of variance test was used to explore the relationship between the RSVs during these time periods.

Additionally, various internet search engines provide information about the absolute number of webpage results available for a search term reported annually, which can provide insight into availability of information to the public. An increase in the number of webpages containing a search term year to year indicates an increased public interest in that term; thus, we examined the annual number of websites containing “morcellation” using the search engine Bing for a 3-year period from 2011 to 2014 (the 3 years prior to the communications), 2014 (the year of the communications), and 2014 to 2017 (the 3 years following the communication). All statistical analyses were performed using Stata version 13.1 (StataCorp LLC).

## Results

**Figure 1:**
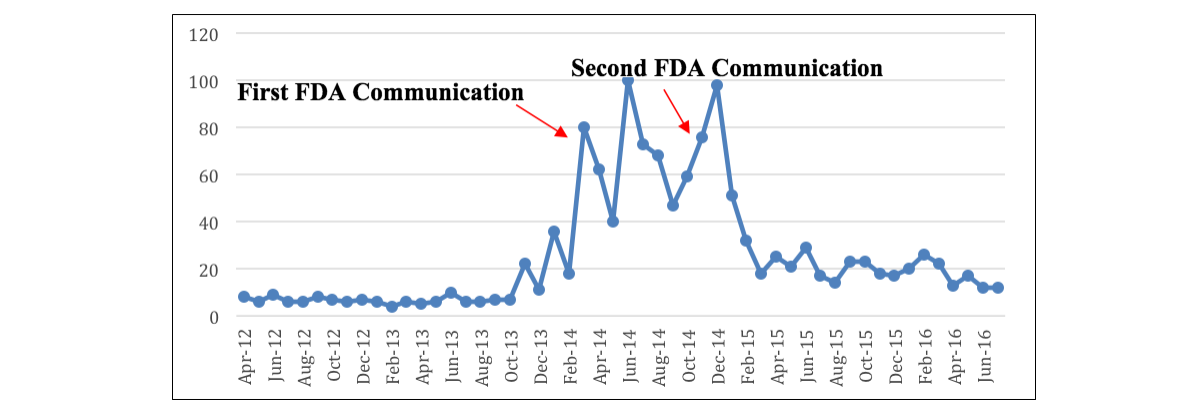
Weekly relative search volume from Google Trends for the term "morcellation."

The mean RSV prior to the initial 2014 FDA safety communication was 12, indicating very little baseline search volume for uterine morcellation (Table 1). This significantly increased to an average of 60.3 in the year following the FDA safety communication and decreased to 19.3 thereafter (*P*<.001). A peak RSV of 100 occurred twice, once in June 2014, between the 2 communications, and again in December 2014, after the second FDA safety communication (Figure 1).

**Table 1:** Weekly relative search volume for the term “uterine morcellation.”

Time period	Relative search volume, mean (SD)	*P* value
Prior to US Food and Drug Administration communication	12.0 (15.8)	<.001
One year after communication	60.3 (24.7)	<.001
Thereafter	19.3 (5.2)	<.001

**Table 2:** Number of webpages containing the term “morcellation.”

Time period	Number of webpages
2011	10,800
2014	18,880
2017	36,200

**Figure 2:**
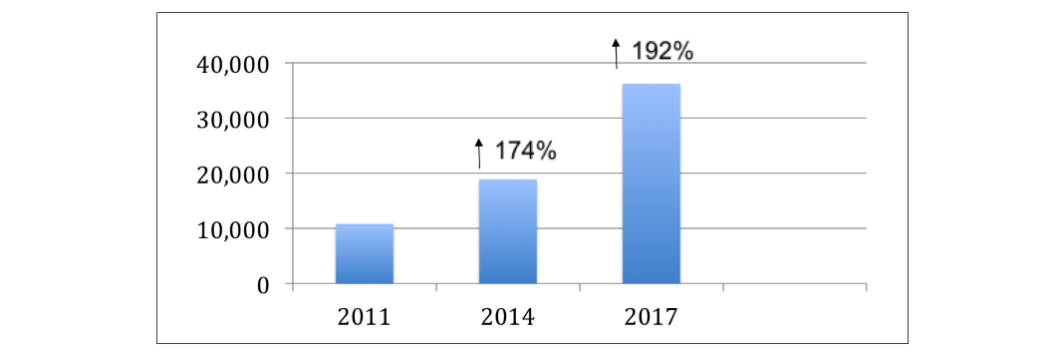
Webpages containing the term "morcellation."

The mean number of webpages containing the term “morcellation” in 2011 was 10,800 (Table 2). This rose significantly to 18,800 during the year of the communication in 2014, representing an increase of 174% (Figure 2). The largest increase in the annual number of webpages was seen in 2017, when the number rose to 36,200, representing an increase of 192%.

## Discussion

### Principal Findings

Uterine fibroids are common, with up to 80% of women having fibroids by age 50 years [8]. The FDA statements to discourage the use of power morcellation were driven by the risk of undiagnosed uterine malignancy. Ackenbom et al [9] demonstrated that the rate of occult malignancy in patients undergoing hysterectomy for pelvic organ prolapse was significantly lower than in other patient populations. In addition to the risk of cancer progression, morcellation may increase the risk of parasitic leiomyomata and iatrogenic endometriosis [10]. The alternatives to power morcellation include open surgery, extending a laparoscopic incision to remove the specimen, or in-bag morcellation. The use of a bag for morcellation has been shown to increase operative time [11]. Given the low risk of malignancy, it would be difficult to demonstrate that in-bag morcellation would result in a decrease in cancer risk, and no evidence for this is currently available.

As technology continues to evolve, patient awareness of publicly available health information becomes increasingly important for physicians to consider when counseling patients. The use of Google Trends has become increasingly important in health care, with a systematic review of over 70 papers showing a 7-fold increase in the number of Google Trends publications from 2009 to 2013 [6]. Matta et al [12] demonstrated that urologists are drastically increasing their use of technology and social media, and patients are likely to seek out health care information both from their physicians and online. Previous authors have used Google Trends to demonstrate that patients in the United States are more likely than those in several other countries to search for topics related to cancer [13] such as uterine power morcellation.

Previous authors have shown an increase in the search term “pelvic organ prolapse” using Google Trends that was associated with the 2011 FDA safety communication [14]. Similarly, we used Google Trends to assess the impact of the 2014 FDA safety communications regarding the use of the power morcellator. This decrease in use may be at least partially driven by patient awareness, as shown by substantial increases in search volume and total number of webpages containing morcellation around the time of the FDA safety communications.

### Limitations

Our study does have limitations. By limiting our analysis to Google Trends and Bing, we do not capture any relevant volume from other search engines. However, Google is the most widely used search engine in the United States. Furthermore, internet users tend to be younger, while patients undergoing hysterectomy are likely to be older. However, use of the internet for medical research is gaining popularity among older patients, with nearly 75% of primary care patients over the age of 65 years using the internet and nearly half using it to access health information [15]. Additionally, decreased use of the morcellator could result from provider-driven decisions and not just patient awareness.

### Conclusion

In summary, the decrease in the use of the power morcellator for uterine specimens at the time of hysterectomy that followed the 2014 FDA safety communications may be related to a significant increase in Google search volume and mean number of webpages containing the term “morcellation.” This suggests that patient awareness may be in part driving the decreased use of uterine power morcellation and may indicate the need for clinicians to expand their scope of preoperative counseling or alter practice patterns and surgical technique.

## Abbreviations

FDA: US Food and Drug Administration
RSV: relative search value
